# The complex origin of *Astyanax* cavefish

**DOI:** 10.1186/1471-2148-12-105

**Published:** 2012-06-30

**Authors:** Joshua B Gross

**Affiliations:** 1 Department of Biological Sciences, University of Cincinnati, 312 Clifton Court, Cincinnati, OH, 45221, USA

**Keywords:** Regressive phenotypic evolution, Cave biology, Genetics

## Abstract

**Background:**

The loss of phenotypic characters is a common feature of evolution. Cave organisms provide excellent models for investigating the underlying patterns and processes governing the evolutionary loss of phenotypic traits. The blind Mexican cavefish, *Astyanax mexicanus*, represents a particularly strong model for both developmental and genetic analyses as these fish can be raised in the laboratory and hybridized with conspecific surface form counterparts to produce large F_2_ pedigrees. As studies have begun to illuminate the genetic bases for trait evolution in these cavefish, it has become increasingly important to understand these phenotypic changes within the context of cavefish origins. Understanding these origins is a challenge. For instance, widespread convergence on similar features renders morphological characters less informative. In addition, current and past gene flow between surface and cave forms have complicated the delineation of particular cave populations.

**Results:**

Past population-level analyses have sought to: 1) estimate at what time in the geological past cave forms became isolated from surface-dwelling ancestors, 2) define the extent to which cave form populations originated from a common invasion (single origin hypothesis) or several invasions (multiple origin hypothesis), and 3) clarify the role of geological and climatic events in *Astyanax* cavefish evolution. In recent years, thanks to the combined use of morphological and genetic data, a much clearer picture has emerged regarding the origins of *Astyanax* cavefish.

**Conclusions:**

The consensus view, based on several recent studies, is that cave forms originated from at least two distinct ancestral surface-dwelling stocks over the past several million years. In addition, each stock gave rise to multiple invasions of the subterranean biotope. The older stock is believed to have invaded the El Abra caves at least three times while the new stock separately invaded the northern Guatemala and western Micos caves. This renewed picture of *Astyanax* cavefish origins will help investigators draw conclusions regarding the evolution of phenotypic traits through parallelism versus convergence. Additionally, it will help us understand how the presence of cave-associated traits in old versus young cave populations may be influenced by the time since isolation in the cave environment. This will, in turn, help to inform our broader understanding of the forces that govern the evolution of phenotypic loss.

## Background

The mechanism governing the evolution of regressive features remains unknown. Regressive features are highly conspicuous in cave-limited organisms, arise frequently across the animal kingdom, and are probably more common than adaptive traits [1]. In recent years, a great deal of attention has returned to the blind Mexican cavefish, *Astyanax mexicanus*, as a powerful model for genomic and evolutionary research. This is a credit to the tractability of *Astyanax* as a laboratory animal and the availability of both surface and cave morphotypes. Until recently, our ability to draw conclusions on fundamental questions pertinent to the evolution of *Astyanax* has remained limited by an incomplete understanding of the origin of these fascinating creatures.

For instance, the origin of a trait through convergent versus parallel evolution rests on a clear understanding of the inter-relationships of distinct cave populations [2]. Hybridization between cave and surface morphotypes in nature may lead to incorrect groupings of fish based on shared allelic backgrounds [2]. Further, the origin of a cave-limited trait, present widely across different cave forms, will be interpreted differently (e.g., through rapid selection versus drift) depending on the estimated time since isolation of cave forms from their surface-dwelling ancestors [3].

Longstanding questions regarding the origin of *Astyanax* cavefish have centered on several topics. One of the earliest questions relates to the number of times the El Abra limestone caves of northeastern Mexico were colonized by ancestral surface-dwelling forms [4,5]. Understanding the number of times the subterranean environment has been invaded can clarify whether cave-associated traits arise through widespread convergence by multiple, independent populations [6,7]. More recently, a great deal of interest has centered on the extent to which gene flow occurs between surface- and distinct cave-dwelling forms [2,8,9].

This work covers two principal topics: 1) a brief review of historical studies in the field of *Astyanax* biology, and 2) a review of recent analyses that have added significant clarity to longstanding questions. By comparing and contrasting these two topics, this review offers a summary of the progression of work in the field of *Astyanax* biology and provides a framework for understanding competing hypotheses. This review aims to provide a heuristic tool through which *Astyanax* researchers can better evaluate the evolutionary mechanisms leading to regressive phenotypic loss.

### Geological and climatic factors affecting *Astyanax* origins

**Figure 1:**
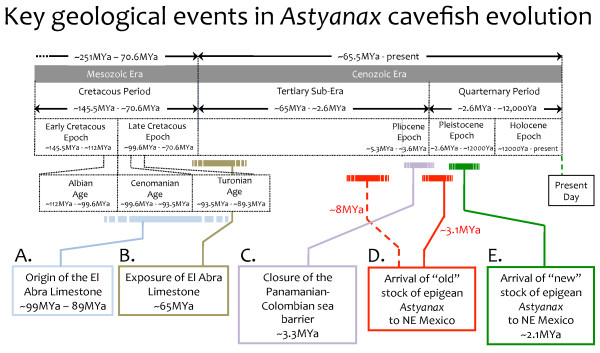
**Numerous geological events have influenced the settlement of*Astyanax*cavefish in northeastern Mexico.** The El Abra Limestone formation materialized from the vast deposition of marine sediment during the mid-Cretaceous Period (**A**; light blue). The El Abra Limestone gradually became exposed at the surface ~65 MYa (**B**; brown). Over the course of several millions of years, a vast network of limestone caves evolved in the area of present-day northeastern Mexico. These caves were subsequently invaded by ancestral surface-dwelling *Astyanax* fish. Surface-dwelling forms migrated northward from South America in two waves. The older wave arrived in Mesoamerica either ~8 MYa, via an incipient land bridge (**D**; dashed red line) that connected South and North America, or shortly after (**D**; solid red line) the closure of the Panamanian-Columbian sea barrier ~3.3 MYa (**C**; purple). This older wave seeded the caves of the El Abra region (Figure 2). A more recent wave of new epigean stock colonized the region ~2.1 MYa (**E**; green) and seeded the northern Guatemala caves and the western Micos caves (Figure 2).

**Figure 2:**
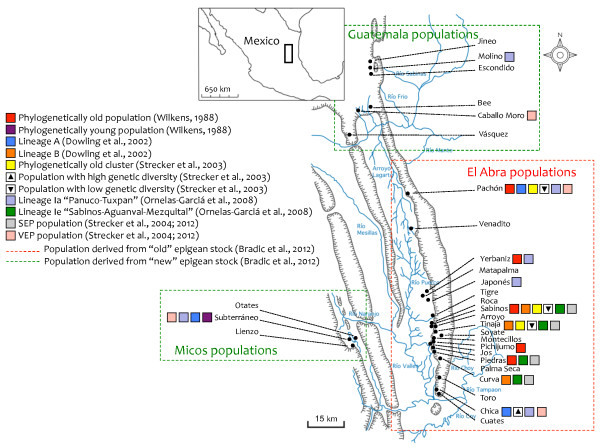
**Summary of population-level analyses in cave and surface populations of*Astyanax mexicanus*.** Over the course of several millions of years, ancestral *Astyanax* (surface) fish repeatedly invaded the limestone karst region of northeastern Mexico (inset). These invasions have led to 29 named populations in the El Abra region, many of which are significantly distant from one another. Several reports have added to our growing understanding of the interrelationships among cave forms. The results of these reports, based on mitochondrial and nuclear gene sequence analysis, are summarized here (colored boxes; see legend). Note that several caves have not yet been sampled (e.g., the northern Jineo and Escondido caves). Classical reports first suggested that recent cave forms may have originated from a single colonization [38], however contemporary reports argue for a much earlier colonization into northeastern Mexico and subsequent colonization of subterranean caves by an “old” (red dashed line) and “new” stock (green dashed line) of surface-dwelling ancestors [7-11,21].

*Astyanax* cave forms evolved in a complex geographical region of present-day Mexico that has represented a transitional zone between the Neotropic and the Nearctic regions since the late Cenozoic era (Figure 1) [10-12]. The Sierra de El Abra is a large carbonate complex, situated to the east of the Sierra Madre Oriental, extending roughly 150 km from the northwest to the southeast (Figure 2) [13,14]. The region of the El Abra caves harboring blind cavefish represents a calcareous reef complex that arose during the middle Cretaceous era (Figure 1A) [13,15,16]. Aguayo-Camargo (1998) analyzed the El Abra Limestone at its type locality (near Ciudad Valles in San Luís Potosí, Mexico) utilizing both petrological and fossil characters. This analysis indicated that the age of the exposed “lower” El Abra Limestone is early Cenomanian (~93.5 to 99.6 millions of years ago (MYa); Figure 1B) [13]. The exposed “upper” limestone is dated to the late Turonian (~89.3 – 93.5 MYa; Figure 1B) [13]. Together, these exposed regions represent two major sedimentary environments, respectively: an older ‘rudist-reef’ environment (termed the Taninul member), and a younger ‘back-reef’ environment (termed the El Abra member). Subdivisions within each region can be identified based on differences in faunal diversity, composition and oil emplacement [13,17]. The unexposed subsurface region is older, dating to the Albian (~99.6 – 112 MYa) [13,18-20]. The El Abra Limestone became exposed in the late-Cretaceous and early Tertiary eras (~65 MYa; Figure 1B), following movements during the Laramide Orogeny [5,16]. Gradual erosion of the limestone bedrock led to formation of a significant cavernous network of subterranean caverns beginning around the mid-Tertiary period [5].

The caverns themselves developed through communication with the terrestrial surface via two routes. Through the process of stream capture, water invaded fractures in overlying impermeable rock, leading to a steep rock face at the cave entryway [16]. The majority of cave formations in the El Abra region harboring cavefish are these sotanós, suggesting that perhaps cave colonization originally occurred through inadvertent trapping of fish into a cave entrance pit (e.g., during spring flooding; see [5]). The second mode of formation involves collapse of a cavernous roof through erosion that unites the cave with the overlying terrestrial environment. These cave formations, cuevas, are distinguished throughout northern Mexico by a distinct geological structure [5].

An important feature of the caves in this region is elevation (i.e., vertical) differences that likely serve as physical barriers (at least at the cave entrances) between cave/surface populations [5]. Indeed, several of the cave pools in this region are “perched” at significant elevations above sea level. Some *Astyanax* cave populations are more isolated than others [5]. For instance, the southernmost Chica cave entrance is located 49 meters above sea level, whereas the Vásquez cave entrance resides 422 meters above sea level [5]. According to Mitchell et al. (1977), the average altitude of cave entrances across the region is ~ 220 meters above sea level [5]. While these authors did not regard any of the cave populations as being “absolutely” isolated, vertical separation at the cave entrance and within the cave, i.e. “perched pools”, provides an obvious spatial isolator between populations [5].

In addition to this vertical isolation, the presence of an internal drainage divide for Río Mante and Río Choy likely serves as a division for populations from the northern and southern El Abra caves, respectively (Figure 2) [5]. A pathway from the southernmost El Abra caves to the northern Pachón cave is believed to have existed in the past [5]. Indeed, Bradic et al., (2012) argue that the Pachón cave was likely established as an independent population from the rest of the southern El Abra cluster of cave populations. This is based on the highly complex geology of underground passages between caves (if they are even navigable) as well as the significant F_ST_ values between the Pachón cavefish and fish from the other El Abra caves (see below)[8]. The authors note that these measures of genetic distance reflect both the current isolation as well as a probable independent origin [8].

An additional geological variable that has likely influenced the settlement of *Astyanax* populations in Central America is the Trans-Mexican Volcanic Belt (TMVB), which harbored intense volcanic activity 3–12 MYa, through the present day [11]. It is believed that the TMVB has served to limit gene flow, however recent studies suggest *Astyanax* populations have crossed this geological barrier at least three times, and that all three migrant populations have invaded the subterranean environment [9,21].

The geological features serving to isolate cave populations have probably influenced the morphological and physiological evolution of the inhabitants. For example, given that Pachón cavefish are perched populations, they encounter far less seasonal flooding, and experience a lower nutrient base [22]. Under these conditions, metabolic changes that favor survival in depauperate caves are predicted to evolve [3,22]. Indeed, an analysis of standard and routine oxygen consumption carried out between surface-dwelling forms and three cave populations indicated significant variability in metabolic rate [22]. The isolated Pachón cavefish population demonstrated the lowest standard oxygen consumption rate (0.230 ± 0.036 mg O_2_ g^-1^ h^-1^; [22]) compared to surface fish (0.314 ± 0.081 mg O_2_g-^-1^ h^-1^). Chica cavefish demonstrated an intermediate standard oxygen consumption rate (0.277 ± 0.063 mg O_2_g^-1^ h^-1^; [22]) which may be explained, in part, by presence of a large bat roost in this cave that has provided a more constant food supply that mitigates (or possibly eliminates altogether) the effects of past bottlenecking events in this population [5,10,22,23].

Geographic distance between cave localities has also likely played a role in past colonization. Bradic et al. (2012) concluded that the western Micos and northern Guatemala populations (Figure 2) likely represent separate invasions into the subterranean environment since these caves are ~90 km apart from one another, separated by a ridge and two open valleys. Additionally, subterranean karst systems are very dynamic; new caves are created, separated, or merged with older ones through time [9]. Thus dispersal of *Astyanax* cavefish, as reported with the cave crustacean *Gammarus minus*[1,24], may have occurred after the gradual formation and erosion of different cave systems. Indeed, the dynamic nature of the karst may explain some patterns of gene flow recently reported among cave and surface fish populations [8]. Any theory seeking to explain widespread convergence on similar traits among *Astyanax* cave populations must account for diverse geological and ecological influences, such as perched pools and lower food availability, which vary by population.

### Arrival of ancestral epigean forms to Mesoamerica

*Astyanax* is a New World, freshwater fish genus that originated in South America. The absence of a land bridge has long been regarded as the principal limitation to northern invasion of this fish into Central America [25]. It has also been assumed that northward migration of ancestral *Astyanax* surface-dwelling forms followed formation of the Panamanian-Colombian land bridge in the late Tertiary, roughly 3.3 MYa (Figure 1, D solid red line) [5,10,25-28]. A recent study, however, argues that the movement of *Astyanax* forms northward into Central America may have actually occurred much earlier (~7.8 – 8.1 MYa), migrating via an incipient land bridge that existed well before closure of the Panamanian-Columbian sea barrier (Figure 2C, D dashed red line) [11].

If the ancestral forms arrived into Mesoamerica at some point between ~3.1 MYa and 8.1 MYa, when did these fish actually invade the caves? Early estimates of cave colonization were coarse. For instance, Avise and Selander (1972) suggested troglobites colonized caves prior to the end of the Pleistocene era – between ~2 million and ~10,000 years ago. This timing was extrapolated from work performed in temperate zone caves in which cave animals were argued to have become isolated during a warming trend of Pleistocene inter-glacial events [3,29,30]. Thus, if using the Holocene as a minimum time-since-isolation, subterranean forms in temperate caves may have been isolated as recently as ~10,000 years [3].

But, what about aquatic tropical caves? Evidence suggests that the tropics experienced multiple glacial/inter-glacial phases throughout the Pleistocene [31,32]. Further, in contrast to non-aquatic organisms, aquatic species in tropical caves are assumed to have been isolated for a much longer period of time [33]. For instance, Hobbs and Barr (1960) argued the cave-adapted crayfish *Orconectes* has likely been isolated from its ancestral surface counterpart for ~2 MYa [34].

Later studies in *Astyanax* estimated that the timing of ancestral invasions into the caves ranged from the late Pliocene [15] to the late Pleistocene [5,25,27,35,36]. A series of recent studies, however, have suggested that the latter estimate is far too recent. An earlier estimated time of invasion into the cave environment comes from recent work suggesting certain caves were colonized quite early by marine animals (*Speocirolana bolivari, Troglocubanus perezfarfanteae* and *Spelaeomysis quinterensis*; [10]). The most recent view, based on consensus drawn from several reports, is that two ancestral waves of surface-dwelling stock invaded the region of the caves. An older stock of surface fish invaded the caves of the El Abra region (see below; Figure 1D) [8], and a younger stock subsequently invaded the western Micos caves and the northern Guatemala caves (see below; Figure 1E) [8]. Of course, the precise timing of invasions into the caves by surface-dwelling forms is unknown. However, tilapia fish have been noted in certain El Abra caves (e.g., Yerbaniz; [8]). Since these fish were only introduced to Mexico in the 1980s [37], in principle surface-dwelling fish are capable of invading the subterranean environment remarkably quickly [8].

## Single versus multiple origins

### Single origin hypothesis

How many invasions of the underground environment led to the extant distribution of *Astyanax* cave populations? Breder and Rasquin (1947) proposed that surface form fish entered subterranean caves via the Río Tampaón into the Chica cave, and dispersed northward via cavernous subterranean connections below the Valle de Antiguo Morelos to the Sabinos and Pachón caves [38] (reviewed in [5]). This hypothesis was popularized, and largely accepted in the literature, despite the fact that the authors provided two alternatives, including: 1) origin of each cave form near their present geographical position (i.e., multiple origins), and 2) a single origin at the northern caves followed by southern migration to the Chica locality [38].

Alvarez (1946) tested the single-origin hypothesis through assessment of a number of morphological features (e.g., fin ray number, body proportion measurements) in the Chica, Sabinos and Pachón caves. This work demonstrated overlapping population-level measurements that bridge from the Chica to the Sabinos to the Pachón caves [39]. Later, a hybrid analysis by Şadoğlu (1957) demonstrated that crosses between surface forms and Chica, Sabinos and Pachón cavefish resulted in progressively smaller eyes in offspring. This experiment supported the notion that more isolated cave forms (i.e., Pachón compared to Chica) demonstrate more significant regressive loss [40]. However, a subsequent analysis by Wilkens (1970) failed to observe noticeable differences in eye size or structure in Pachón and Sabinos cavefish [41]. Moreover, classical studies did not account for the fact that Chica represents a possible “hybrid” population [25] that experienced recent gene flow from the nearby epigean fish [5].

The single origin hypothesis was supported, in part, by the findings of Avise and Selander (1972) who found low levels of heterozygosity in three populations of cavefish (average heterozygosity = ~7.7%) compared to six populations of surface fish (average heterozygosity = ~11.2%). Based on this evidence, the authors remarked, “The eyeless, unpigmented condition is believed to have evolved in whole or part prior to the present-day subdivision of the populations,” (p.16 [16]). While the number of individuals sampled from each cave was large, only three of the now 29 known populations were represented in this analysis [7].

Today the single-origin hypothesis has been dismissed for a number of reasons. First, as Mitchell et al. (1977) argued, ancestral surface forms likely invaded the limestone caves several times. This assumption was based on the presence of geographic barriers between various caves (e.g., mountainous areas between the Micos and El Abra regions) that renders a single origin unlikely [5,7,42]. Secondly, contemporary population-level analyses have offered very strong support for multiple invasions into the cave environment through extensive intra- and inter-population variation analyses (see below) [2,7-11,21,23,24]. Finally, complementation crosses carried out between members of different cave populations demonstrate that several cave-associated traits arise through different genetic loci in different populations. If geographically distinct cave forms arose from the same eyeless founder population, then all offspring would be predicted to harbor eyeless phenotypes governed by the same genetic loci. This is not the case – an analysis of eye phenotypes in F_1_ individuals [15,43]; demonstrates distinct genetic bases account for the loss of the visual system in different cavefish populations [43,44]. Since distinct genetic changes account for the eye loss phenotype in different cave populations, this suggests each cave population evolved this trait independently.

### Multiple origin hypothesis

The notion that cave forms arose through multiple colonizations, each followed by short-range dispersals, gained favor as more cave populations were discovered. From the mid-1940s through the 1970s, cave expeditions in northeastern Mexico led to discovery of 26 additional caves – many separated by distinct geological barriers (Figure 2). Categorical distinctions in the literature contrasted “phylogenetically young” and “phylogenetically old” populations based on the degree of troglomorphy evident within cave populations (see Figure 2). Cave forms demonstrating mild cave-associated phenotypes (e.g., reduced but not absent pigmentation, visual system regression but not total loss) were regarded as “young” populations [42]. More ancient cavefish populations were those demonstrating more extreme phenotypic changes, e.g. complete loss of the visual system structures and significantly enhanced “constructive” morphologies to facilitate identification of nutrition in the trophic-poor context of the cave.

 Wilkens (1988) categorized certain cave populations as “phylogenetically old” populations – including Pachón, Sabinos, Piedras, Yerbaniz and Pichijumo. Other caves, including the Micos cave populations (Otates, Subterráneo, Lienzo), represent *in sensu* or “phylogenetically young” cave populations (Figure 2) [42]. The designations adopted by Wilkens (1988) were based on population-level characteristics, e.g., size of the eye rudiment is ~10-20% of the size of surface form fish. An additional categorical description of ‘hybrid fish’ refers to a recent surface x cave form introgression event, e.g. the Chica cave [24].

Phylogenetically “old” versus “young” categories were based on the notion that the accumulation of neutral mutations is the principle mechanism driving regressive evolution, and therefore a longer time since isolation is predicted to lead to the accumulation of more phenotypic regression [3]. However, the precise mechanism(s) driving the evolution of regressive traits remains unknown. A recent study argues that cave-associated phenotypes are selected for in the cave environment, given the level of gene flow that has been observed between cave and surface-dwelling populations [8]. In light of this gene flow, the cave phenotype remains highly stable in different populations implying that cave-associated alleles are under strong natural or sexual selection [8]. Irrespective of the mechanism that governs evolutionary loss of phenotypic characters in the cave environment, the origin of *Astyanax* cave forms from multiple invasions and colonization events is now well accepted. Supporting evidence from population genetic studies is presented below.

### Population level analyses

Recent population level analyses have provided clarity to early classifications of “phylogenetically old” versus “young” cave populations. These studies estimate the age of different cave populations utilizing genetic rather than strictly morphological characteristics. This is particularly important given that widespread convergence on the cave phenotype, through diverse genetic mechanisms, renders morphology a less powerful tool in defining relationships among different cave forms [11].

The first analysis of mitochondrial DNA (mtDNA) sequence variation was carried out using the *NADH dehydrogenase 2* (*ND2*) locus [7]. This study found the level of mtDNA divergence observed in *Astyanax* is both high, and comparable to other freshwater fish species [7]; see also [21]. Combining genetic sequence analysis with morphological variation data (rib number), the authors discovered that cave forms cluster into two lineages (A and B; Figure 2). Both lineages harbor eyeless fish populations, providing support for eye loss having evolved multiple times (Figure 2), a finding consistent with results of complementation studies (see above). The majority of lineage A fish have 12 ribs, including fish from the Subterráneo, Pachón and Chica caves along with surrounding surface fish. The authors suggested that these cave forms originated more recently from surrounding epigean populations [7].

The majority of individuals in Lineage B (Sabinos, Curva and Tinaja cavefish) harbor 11 ribs [7]. These populations demonstrated no similarity to other cave forms or the surrounding epigean populations. The authors argued that these cave forms were established by an ancestral surface-dwelling form that is either extinct, or no longer present in this region of Mexico [7]. Indeed, these lineages are more similar to another species from southern Mexico and Costa Rica (*Astyanax aeneus*) than they are to each other. The authors found that surface fish harbor more genetic variation compared to cave forms. However, more than one haplotype was observed in both the Tinaja and Subterráneo caves. This may be due to the fact that Tinaja represents the largest cave, and Subterráneo harbors an opening through which epigean forms may enter, and therefore could be explained by introgression with the surface fish. The authors concluded that the divergent ND2 haplotypes observed between lineages is consistent with multiple origins, followed by convergence on the eye loss phenotype [7].

A subsequent analysis of genetic variation within cave forms was carried out using nuclear (microsatellite) loci as well as mtDNA [23]. This study reported little gene flow between cave and surface forms, and that every cave population except the Chica population demonstrated extremely low microsatellite variability, likely caused by periodic bottleneck events in the past [23]. These authors first noted important disagreements between mtDNA and microsatellite data. Based on microsatellite data, Pachón, Tinaja and Sabinos cluster together as “phylogenetically old” populations. In contrast, based on mtDNA sequences, Tinaja and Sabinos group together – while Pachón and Chica group together along with surrounding surface populations (Figure 2) [23].

These results, largely congruent with the findings of Dowling et al. (2002), implied a common origin for the Pachón, Sabinos and Tinaja caves. Pachón cavefish, which harbor very low levels of genetic diversity, may have been isolated from ancestral surface forms for a much longer period of time compared to the other cavefish populations [23]. The Chica cave, in contrast, was concluded to be an ancient cave population that subsequently hybridized with a surface population. This recent gene flow is demonstrated by reduced divergence between Chica and surface form fish [23]. In sum, these two reports first suggested multiple origins for distinct cave populations, via at least two independent invasions [7,23].

This analysis was followed by a phylogeographic analysis of surface and cave forms based on variation in the mitochondrial gene *cytB*. This extensive study, which sampled 174 individuals comprising 9 cave and 26 surface fish populations, identified seven major clades. The observed clade distribution is likely due to episodes of invasion followed by extinction of surface forms, as reported also by Dowling et al., 2002. Four cave localities (Piedras, Curva, Sabinos and Tinaja) were categorized as “strongly eye- and pigment-reduced populations” (SEP) that fail to cluster with surface fish near surrounding regions (Figure 2). The ancestors of this population were argued to have survived as “thermophilic relics” during climatic changes of the Pleistocene when surface ancestors went extinct [10].

This analysis also described a “variable eye- and pigment-reduced” (VEP) clade based on mtDNA. This clade, similar to Lineage A [7], clusters with surface fish from the surrounding area [10]. Based on the high degree of genetic divergence among the different clades, these authors concluded that surface fish re-invaded rivers, streams and caves from the south. The only phenotypic departure from these descriptions is Pachón which groups with VEP, however demonstrates troglobitic phenotypes more similar to the SEP clade. The authors explain this as due to introgressive hybridization with surface forms in the past [10].

In sum, the SEP cave populations are older, having been derived from the first epigean fish invasion [10]. In contrast, the VEP population cluster with surface forms from the same geographic region, suggesting a more recent invasion. This report used a divergence estimate, based on *cytB* sequence variation (1.5%/MY; [45]), to estimate a time of divergence between surface and cave forms of 1.8 – 4.5 MYa (average = ~3.1MYa; Figure 1D solid red line). This timing follows the closure of the Panamanian-Colombian land bridge, allowing northward migration from South America into Central America (Figure 1C) [27]. Following the initial invasion, surface forms are hypothesized as having gone extinct as a consequence of surface cooling during the Pleistocene/late Pliocene [46]. The SEP populations survived in warm subterranean waters [46] in refugia. A second invasion, seeding the more recently colonized caves, then likely spread northward ~1.8 – 3.0 MYa (average = 2.1MYa; Figure 1E).

A population analysis that followed this report, however, argued for an earlier invasion of surface form fish into northeastern Mexico [11]. These authors performed an extensive population genetic analysis of the entire *Astyanax* genus in Mesoamerica by examining DNA sequences of three mitochondrial genes (*cytBCOI16 S*) and a nuclear gene (*rag1*) using 208 individuals from 147 localities [11]. This analysis yielded six major phylogenetic groups – *Astyanax* cave forms fell into Group I, subdivided into four clades. Of the four lineages within Clade I, the more ‘recent’ troglobitic forms are in lineage Ia (“Panuco-Tuxpan”, comprising cave populations from the Huastecan region: Chica, Molino, Micos (i.e., Subterráneo), Pachón, Yerbaniz, Japonés). More ancient populations are clustered within clade II (Lineage Ie, “Sabinos-Aguanaval-Mezquital”), including cave populations from the Sabinos cluster, comprising Curva, Tinaja, Sabinos, and Piedras (Figure 2). This finding is consistent with the conclusion that Sabinos and Tinaja cavefish are remnants of an early *Astyanax* invasion into Mexico [10,21].

One of the principal findings of this report was the observation of a high degree of taxonomic diversity in *Astyanax* fish compared to other studies. The authors attributed this diversity to a much earlier colonization of *Astyanax* into Mesoamerica. Congruent with other studies, this report argued for multiple (at least two) independent colonizations of epigean forms into upper Mesoamerica. The first colonization of epigean forms was predicted to be quite ancient (~7.8 – 8.1 MYa; Figure 1D dashed red line). This earlier timing implies the presence of an ‘incipient’ land bridge that permitted migration of *Astyanax* fish northward *prior* to closure of the Panamanian-Colombian landbridge (Figure 1C, D dashed red line) [11]. This timing of origin for cave forms is much earlier than previously suggested (~3.1MYa) [10]. While these authors estimated divergence times using *cytB*, the discrepancy between their estimates and others (see [10]) is a consequence of different calibration points (i.e., rising of the Sierra of Perija and Merida Mountains and final closure of the TVMB) and dating estimates (using an average of ~0.8%/million years; see [11,47,48]). Their earlier estimate of colonization into northeastern Mexico, however, is consistent with the proposed timing of invasion of other species into Mesoamerica. For instance, the catfish *Rhamdia laticauda* also reportedly invaded this region prior to closure of the Colombian-Panamanian land bridge [49].

A more recent study, evaluating both mtDNA and microsatellite data in cave and surface populations of *Astyanax*, further characterized the reported discordance between nuclear genotypes and the mtDNA clades previously defined from other studies. Hausdorf et al., (2011) surveyed variation at six microsatellite loci in 25 populations comprised of 4 cave and 21 surface-dwelling localities. The results of their mtDNA analysis were largely congruent with prior findings [11,23], however nuclear genotypic data revealed two clusters. Cluster I comprised most of the surface populations in the region of northeastern Mexico and most of the individuals from the Chica cave. Cluster II, the “old” cave cluster, included cavefish from Pachón, Tinaja, and Sabinos as well as the remaining fish collected from the Chica cave (~14% of their collected samples).

Admixture analyses indicated that there has been recent gene flow between distinct mitochondrial clades, most notably from the Pachón cave locality [21]. The authors concluded that the Pachón cave was colonized by the “older” invasion of northeastern Mexico, and that the disagreement between the mtDNA clades and nuclear genotypic clusters can be explained by mitochondrial “capture” that occurred between Pachón and a neighboring surface population [21]. These authors also suggest that the evolution of “constructive” traits in *Astyanax* cavefish (e.g., enhanced taste and lateral line senses), that have evolved independently in multiple cave populations may have resulted in pre-mating isolation, representing an initial step towards parallel speciation [21].

To determine if *Astyanax* cavefish represent an instance of parallel evolution, Strecker et al. (2012) performed a population-level analysis of seven cave and seven surface populations. These authors reported significant population differentiation between *Astyanax* cave and surface forms through analyses of both nuclear (microsatellite) DNA and mtDNA. Their mtDNA analysis also agrees with findings of other groups indicating epigean *Astyanax* invaded northeastern Mexico, and the caves, at least twice [7,10,11,21,23].

In this study, the old invasion was distinguished on the basis of mtDNA (clade G = old) [23], whereas the new invasion corresponds to clade A, which is more similar to southern *Astyanax* surface fish. Two nuclear genotypic clusters were distinguished from these old and new invasions [21,23]. As observed from prior studies, Pachón cavefish group with the old invasion based on nuclear genotypic data, but group with neighboring surface populations and the Chica cave based on mtDNA sequence similarity (Clade A) [21,23], further supporting mitochondrial capture of the surrounding surface fish by the “old” Pachón cave population [21,23]. Interestingly, in this study disagreements between mtDNA clade assignment and nuclear genotypic clusters were observed in more *Astyanax* cave populations (Yerbaniz, Molino, Pichijumo, Caballo Moro) implying mitochondrial “capture” may be a frequent phenomenon in *Astyanax* cavefish [9].

Using a STRUCTURE analysis of microsatellite data [9], the authors assigned their groups to each of five clusters. Cluster I includes all surface fish analyzed from northern Mexico along with surface fish collected from the Yerbaniz cave and a single individual from each of the Micos and Chica caves. Cluster II includes all of the fish from the “old” cave invasion, including Tinaja and Pachón cavefish, all blind fish collected from Yerbaniz cave, and all but one fish from the Sabinos and Chica caves. Cluster III includes one fish from the Micos cave, two fish collected from Río Coy, and most fish from two surface populations in southern Mexico. Cluster IV includes all of the Caballo Moro cavefish. Cluster V includes most fish from the Micos and Chica caves, one surface fish from near the Pachón cave entry, four surface fish from near the Micos cave locality, one fish from Sabinos cave, and three surface fish from the Río Coy and Malatengo localities. Based on these analyses, the authors concluded that little (if any) gene flow occurs between the cave populations and their neighboring surface populations.

In contrast, a very recent study by Bradic et al., 2012, analyzing 26 different loci (microsatellites) in 11 cave and 10 surface populations, argues that measurable gene flow has occurred between both cave and surface and from cave to cave populations. This population analysis agrees with prior reports suggesting cave populations originated from two (‘old’ and ‘new’) waves of ancestral surface-dwelling forms [7,9-11,21,23]). The “old” caves are those of the El Abra region (Pachón, Yerbaniz, Japonés, Arroyo, Tinaja, Curva, Toro and Chica). The young caves include those from the Guatemala region to the north (Molino and Caballo Moro), and the western Micos caves (Subterráneo). Certain cave populations (Pachón, Curva, Molino and Caballo Moro) demonstrated the highest levels of genetic differentiation from surface-dwelling fish, likely as a consequence of isolation since these caves are perched populations or isolated from nearby surface streams [8].

This study found cavefish populations are more genetically differentiated than their surface form counterparts, despite surface fish being more geographically separated. This was supported by the finding of lower allelic diversity in cave versus surface populations. The authors also argue that the observed lower genetic diversity, which correlates with smaller effective population sizes, likely arose as a consequence of limited food and space in the subterranean environment and/or periodic bottleneck events [8,23].

In sum, according to Bradic et al. (2012), the entire geographical extent of northeastern Mexico harboring blind *Astyanax* cavefish populations were colonized by an old and new stock of surface-dwelling ancestors. The older population invaded the El Abra caves at least three times and then went extinct (Figures 1D and 2). The younger epigean stock colonized the Micos and Guatemala regions much later, through at least two invasions (Figures 1E and 2). This more recent invasion was established from a wave of ancestral surface-dwelling fish that are closely related to the surface-dwelling forms found in the region today. Based on the independent acquisition and stability of cave-associated traits, despite significant gene flow (see below), these authors conclude that these traits are likely maintained under natural or sexual selection [8].

The consensus view, drawn from several recent population genetic analyses, is that *Astyanax* cave populations originated from two waves of ancestral epigean forms from the south. The older wave gave rise to the El Abra populations, while the younger wave gave rise to the Guatemala and the Micos populations (Figure 1D, E and 2) [8]. Rather than a colonization time on the order of tens of thousands of years ago, as estimated from earlier studies [3,5], the caves were colonized several millions of years ago (Figure 1 and 2) [8,10,11]. Further, rather than a single invasion originating from one ancestral stock, extant cave forms originated from an “old” and “new” stocks of surface-dwelling ancestors [9,10], each through multiple (i.e., at least five) independent invasions [8].

### The origin of Chica cavefish

The precise origin of the southern Chica cave is interesting. This population demonstrates a higher level of genetic diversity compared to most caves, probably as a result of a higher amount migration from surrounding surface fish [8]. Haudorf et al. (2011) explain the variability in eye size among individuals within the Chica cave as caused by periodic hybridization events in the past between Chica cavefish and surrounding surface populations. However, based on the high F_ST_ values observed between cavefish and surrounding surface fish populations, the authors argue that no continuous gene flow occurs from surface fish into the cave [21]. The authors also report that despite the presence of hundreds of surface fish that get swept into two other cave localities (Micos and Yerbaniz) during spring flooding, no appreciable hybridization occurs [21].

Based on this evidence, Hausdorf et al. (2011) concluded that Chica cavefish probably did not arise from the same invasion as the older Sabinos, Tinaja and Pachón caves since most of the fish at this locality grouped with nuclear genotypic cluster I. It should be noted however, that ~14% of the fish from Chica did group with the older caves. These authors argue that Chica was colonized by a younger cave population that later introgressed with an older cave population [21,23].

In contrast, Bradic et al. (2012) concluded that Chica was seeded by the old stock of epigean fish. This was based on the finding that the genetic distance for Chica cavefish was equidistant between the older and newer populations [8]. The results of their F_ST_ analysis demonstrated that this population is significantly different from the other cave and surface populations surveyed in their study. Moreover, the authors suggest that – due to its southern geographic position – Chica may have been one of the first *Astyanax* cave populations seeded from the older wave. This may be due to the fact that Chica harbors a large bat roost that provides a significant nutrient base. This higher nutrient base may have allowed periodic surface migrants to survive in the otherwise nutrient-poor environment of the cave [8].

### Genetic variability within caves and gene flow between populations

Beyond the Chica cave, the level of genetic variability within and between other cavefish populations has long been of interest to *Astyanax* researchers. Generally, cave organisms are assumed to harbor low levels of genetic variability [16]. Classical theories aimed to determine what events lead to reduced levels of genetic variability in cave populations. Soulé (1971) argued that relatively low levels of genetic variability should be observed and maintained in cave-dwelling organisms [50]; reviewed in [16]. On the other hand, Barr (1968) argued that low levels of heterozygosity would be predicted following a cave invasion, but that heterozygosity would subsequently re-expand [51]. Poulson and White (1969) argued that low levels of variability would remain constant – less as a consequence of population bottlenecks or inbreeding - but due to strong stabilizing selection for cave-adaptive traits [52].

To assess the validity of these competing theories in *Astyanax*, Avise and Selander (1972) first set out to determine if cave forms harbored reduced heterozygosity compared to surface form fish. They discovered that cave forms demonstrated reduced levels of heterozygosity (e.g., all 17 loci in Pachón cavefish were monomorphic) compared to surface forms. The common assumption that all *Astyanax* cavefish populations harbor low levels of genetic diversity, however, may need to be revised. Based on an analysis of RAPDs and 19 microsatellite markers, Panaram et al. (2005) demonstrated a low, but not absent, level of genetic diversity in cave populations. After testing the effects of a variety of causes, only the presence of eyed fish within the cave population was associated with increased genetic diversity. The authors concluded that this intra-cave diversity likely reflects recent gene flow between surface and cavefish. Microsatellite analyses provided further support for the notion of measurable gene flow between cave and surface forms [2].

A fascinating case study of intra-cave phenotypic variability occurs at the Caballo Moro locality that harbors both eyed and eyeless fish populations. This cave has a karst “window”, originating from a collapsed roof that illuminates a cave lake during certain times of day [5]. Espinasa and Borowsky (2000) observed the distribution within the cave is non-random with eyed and eyeless fish remaining distinctly within the illuminated and dark portions of the lake, respectively. This division is the result of aggression towards eyeless fish swimming into the lighted region of the cave [53]. These attacks may provide a mechanism by which eyed and eyeless individuals remain subdivided within the same population. RAPD genetic analyses revealed the eyed Caballo Moro fish are more closely related to the eyeless fish than they are to surrounding surface fish populations. This leads to the intriguing possibility that eyed forms were re-established from standing variation in the eyeless population, after the karst window was established [53].

In a more recent study, Strecker et al., 2012 observed that all of the fish at the Caballo Moro locality grouped with a nuclear genotypic cluster (IV) that is more genetically similar to surface fish populations south of the TVMB than neighboring surface populations in northern Mexico. The authors categorized these fish as a VEP (variable eye and pigmentation) population. Based on their microsatellite data analysis, these authors concluded that Caballo Moro cavefish arose from a relatively recent colonization across the TMVB into the cave environment. Thus, Caballo Moro is likely a young cave population that has undergone divergent selection within the cave that has led to the first stages of genetic separation, representing an example of incipient speciation [9].

In other caves, intra-cave variation could be explained, in principle, by differences in trophic diversity or hybridization with surface form fish [10]. The consensus view among several reports is that hybridization with surface fish likely explains phenotypic and genotypic diversity at the Chica cave locality (see above). But what about other cave localities inhabited by *Astyanax* cavefish? Hausdorf et al. (2011) argue that, despite flooding events that bring hundreds of surface fish into the western Micos cave and the Yerbaniz caves, there is no appreciable level of hybridization. Indeed, in Yerbaniz, hundreds of fish are reportedly swept into the caves following spring flooding, however only two specimens have been reported that bear intermediate phenotypes [5,9]. The authors suggest that this failure to hybridize likely occurs as a consequence of competition for limited food supply among surface forms that get swept into the cave. A similar circumstance exists at the Micos cave locality where surface fish present in the cave have been observed to be in a starvation state [54].

Past expeditions to the Pachón cave have similarly reported changes in population-level phenotypes (e.g., from predominantly pigmented to predominantly albino fish) suggesting that contact with surface fish is a random event that occurs unevenly over time [55]. Recent QTL studies carried out by Protas et al., (2006) and Gross et al. (2009) offer evidence of gene flow between caves. Both studies reported loss-of-function alleles associated with pigmentation phenotypes (albinism and *brown*, respectively) from the Pachón cave populations were present in the geographically distant Japonés and Yerbaniz caves [6,56]. These observations suggest that recent gene flow, or presence of the same alleles in the ancestral epigean forms that originally colonized the three cave populations, may explain why the same alleles are observed in geographically distant populations. A clear understanding of the level of gene flow between cave and surface forms, or between different cave populations, is critical for understanding how and why alleles may be shared among different populations.

Strecker et al. (2012) reported no gene flow between the strongly eye- and pigment-reduced (SEP) cave populations (Sabinos, Tinaja and Pachón) and surrounding surface-dwelling forms. Gene flow was not observed even in cases where surface fish were present within the same cave (e.g., the Yerbaniz locality). The authors also reported little gene flow, if any, between populations demonstrating variability in eye size and pigmentation (VEP = Micos, Chica and Caballo Moro; Figure 2) and neighboring surface-dwelling fish. Based on this, the authors argue the VEP cavefish more likely evolved from a recent colonization, rather than from an ancient cave population that subsequently hybridized with surface-dwelling forms.

In contrast, Bradic et al. (2012) reported a significant level of gene flow among *Astyanax* fish. In general, surface populations demonstrated higher rates of migration with other surface populations in a symmetrical fashion. Migration rates between cave and surface populations were generally found to be asymmetrical with more surface migration into the cave rather than from the cave into the surface [8]. The Micos cave population, however, demonstrated a nearly equal amount of migration with the surrounding surface fish population. Migration rates among caves were found to be very low except for caves that were geographically close to one another [8]. This finding suggests that there may be subterranean connections that unite different caves to one another, allowing for the observed (albeit low) frequency of gene flow. An additional factor that may play into this migration is altitude since the authors observed a trend in migration rates that followed gene flow from higher populations (e.g., N2 at 175 m above sea level) to lower populations (e.g., N1 at 125 m above sea level) [8].

## Conclusions

In recent years, through numerous genetic and molecular analyses, a clearer picture of *Astyanax* cave form origins has emerged from the literature. There are important challenges associated with generating a conclusive phylogeny of cave forms since widespread convergence among cave forms renders identification of precise lineages problematic [11]. The consensus view, based on all estimates, indicates that the origins of cave forms are far older than initially assumed. Rather than evolving from a single colonization of the subterranean biotope, several invasions stretching back several millions of years led to the extant distribution of *Astyanax* cavefish populations. Several independent invasions from each “stock” of these epigean ancestors have seeded multiple caves. For instance, both the Pachón and Sabinos cave populations are derived from the same “old” stock [8], but these populations likely invaded each cave at distinct times in the past.

In addition, genetic studies indicate a larger amount of genetic variability within caves exists compared to early estimates. This may indicate a larger relative amount of gene flow both between caves and between surface and cavefish [8]. This observation is supported, in part, by recent QTL analyses that demonstrate the presence of identical haplotypes for *Oca2* and *Mc1r* in geographically distant caves (Pachón and Yerbaniz/Japonés caves; [6,56]).

The growing interest in *Astyanax* cavefish, as a model for understanding regressive phenotypic evolution, underscores the importance of a higher resolution phylogeny for interpreting experimental studies. In addition, studies investigating the genetic basis for trait evolution in cavefish can be useful for informing the extent to which alleles are shared between geographically distinct cave forms.

## Competing interests

The author declares no competing interests.

## Authors’ contributions

JBG wrote the manuscript.
